# Long Stent Implantation on the Left Anterior Descending Coronary Artery at a Follow-Up of More Than Five Years

**DOI:** 10.3390/jcm13010210

**Published:** 2023-12-29

**Authors:** Alessandro Sticchi, Concetta Tatali, Massimo Ferraro, Arif A. Khokhar, Alessandra Scoccia, Alberto Cereda, Marco Toselli, Francesco Gallo, Alessandra Laricchia, Antonio Mangieri, Francesco Grigioni, Gian Paolo Ussia, Francesco Giannini, Antonio Colombo

**Affiliations:** 1 Cardiac Catheterisation Laboratory, Cardiothoracic and Vascular Department, Azienda Ospedaliero Universitaria Pisana, Via Paradisa, 2, 56124 Pisa, Italy; sticchialessandro@gmail.com; 2 University of Pisa, Lungarno Antonio Pacinotti, 43, 56126 Pisa, Italy; 3 Cardiovascular Department, Campus Bio-Medico University Hospital of Rome, 00128 Rome, Italy; coli9_5@hotmail.it (C.T.); f.grigioni@policlinicocampus.it (F.G.); gpussia@hotmail.com (G.P.U.); 4 Department of Interventional Cardiology, EMO-GVM Centro Cuore Columbus, 20145 Milan, Italy; ferraro.emogvm@gmail.com (M.F.); ac84344@gmail.com (A.C.); 5 Department of Cardiology, Hammersmith Hospital, Imperial College Healthcare NHS Trust, London W12 0HS, UK; arifkhokhar@doctors.org.uk; 6 Department of Cardiology, Erasmus University Medical Center, 3015 GD Rotterdam, The Netherlands; scoccia.alessandra@gmail.com; 7 Cardiovascular Department, ASST Santi Paolo Carlo, 20142 Milan, Italy; 8 Department of Interventional Cardiology, GVM Care & Research Maria Cecilia Hospital, 48033 Cotignola, Italy; marco.toselli2@gmail.com (M.T.); giannini_fra@yahoo.it (F.G.); 9 Cardiology Unit, Ospedale dell’Angelo, ULSS3 Serenissima, 30174 Mestre, Italy; galfra87@gmail.com; 10 Cardiovascular Department, ASST Fatebenefratelli Sacco, 20157 Milan, Italy; laricchia.a@gmail.com; 11 Cardio Center, IRCCS Humanitas Research Hospital, Rozzano, 20089 Milan, Italy; antonio.mangieri@gmail.com

**Keywords:** 5-year FU, very long-term follow-up (FU), PCI, long stenting, left anterior descending

## Abstract

Background: Stent implantation represents the standard of care in coronary intervention. While a short stent implanted on a focal lesion located on the left anterior descending artery (LAD) seems a reasonable alternative to an internal mammary implant, the same for long stents is still debated. Methods: We reported the long-term data of 531 consecutive patients who underwent Percutaneous Coronary Intervention (PCI) with long stents in two highly specialized centres. The main inclusion criteria were the implantation of stents longer than 30 mm on the LAD and a minimum follow-up (FU) of five years. The primary endpoint was mortality, and the secondary endpoints were any myocardial infarction (MI), target vessel and lesion revascularization (TVR and TLR, respectively), and stent thrombosis (ST) observed as definite, probable, or possible. Results: In this selected population with characteristics of complex PCI (99.1%), the long-term follow-up (mean 92.18 ± 35.5 months) estimates of all-cause death, cardiovascular death, and any myocardial infarction were 18.3%, 10.5%, and 9.3%, respectively. Both all-cause and cardiovascular deaths are significantly associated with three-vessel disease (HR 6.8; confidence of interval (CI) 95% 3.844–11.934; *p* < 0.001, and HR 4.7; CI 95% 2.265–9.835; *p* < 0.001, respectively). Target lesion (TLR) and target vessel revascularization (TVR) are associated with the presence of three-lesion disease on the LAD (HR 3.4; CI 95% 1.984–5.781; *p* < 0.001; HR 3.9 CI 95% 2.323–6.442; *p* < 0.001, respectively). Re-PCI for any cause occurred in 31.5% of patients and shows an increased risk for three-lesion stenting (HR 4.3; CI 95% 2.873–6.376; *p* < 0.001) and the treatment of bifurcation with two stents (HR 1.6; 95% CI 1.051–2.414; *p* = 0.028). Stent thrombosis rate at the 5-year FU was 4.4% (1.3% definite; 0.9% probable; 2.1% possible), including a 1.7% rate of very-late thrombosis. The stent length superior to 40 mm was not associated with poor outcomes (all-cause death *p* = 0.349; cardiovascular death *p* = 0.855; MI *p* = 0.691; re-PCI *p* = 0.234; TLR *p* = 0.805; TVR *p* = 0.087; ST *p* = 0.189). Conclusion: At an FU of longer than five years, patients treated with stents longer than 30 mm in their LAD showed acceptable procedural results but poor outcomes.

## 1. Introduction

Finding the most suitable conditions to apply percutaneous interventions (PCIs) versus coronary bypass surgery (CABG) is a field in evolution [1].

Of a particularly great importance are lesions located on the left anterior descending artery (LAD) supplying large myocardial masses [2].

While PCI is practical, effective, and less invasive in most simple lesions, CABG maintains the leading role as the most appropriate revascularization modality in patients with more advanced diseases [3].

Special attention is devoted to lesions located on the LAD due to the large myocardium at risk and the unique value of left internal mammary (LIMA) implantation [4].

The LAD runs on the anterior wall of the heart and its importance is supported by anatomic studies detailing the coronary artery vascular distribution. Other findings derive from pathophysiological evidence due to the great impairment in ejection fraction and the remodelling of the left ventricle following acute myocardial infarction caused by isolated LAD occlusion [2].

The LAD has a high-enough prognostic value to be called in medical history the “Vessel of Life” or the “Widow-Maker” for its power to predict a patient’s survival during myocardial infarction or other cardiovascular adverse events [5].

Stenting long lesions located on the LAD appears to be a safe and effective approach at short-term follow-ups; nevertheless, open questions persist regarding the long-term outcomes, including the possibility of future treatment with LIMA implantation [6]. We cannot dismiss the risk of restenosis considering that the length of the stent implanted is directly related to the need for reintervention to treat intra-stent restenosis (ISR) [7]. In some instances, implanting a long stent may come as a solution for a complication requiring a rapid solution without sufficient commitment to optimization [7].

The relationships between the lesion length, stent length, and long-term outcome needs to be carefully studied, especially in key vessels such as the LAD.

Although drug-eluting stent (DES) introduction and its evolution significantly reduced the rate of stent-related complications, the data available on the long term are still poor and we need further investigations.

## 2. Methods

In this retrospective registry, we included 501 consecutive patients from two highly specialised centres, treated in the period from 1 January 2001 until 30 September 2015. The patients included in this analysis underwent PCI on their LADs requiring a stent longer than 30 mm. We report the clinical follow-up survey conducted from 5 years onwards.

The survey started with a sample of 16,200 patients undergoing PCI in the period indicated above. From this initial sample, we carried out a selection based on criteria relating to the type of lesion and the duration of the FU: PCI performed on the LAD with stents longer than 30 mm, and FU greater than 5 years. Patients with hard events before 5 years were included and censored. Therefore, we reached a sample of 802 patients, 4.95% of the initial sample. and with these patients, we worked to accomplish a proper FU of 66.2%, achieving a final population of 531 patients (see patient inclusion flowchart below). The categorical variable “hyperlipidemia” was obtained from medical records according to the NCEP ATP III definition. The characteristics of complex PCI were analysed in subgroups [8].

The endpoint of the study was to evaluate the mortality outcomes, all-cause and cardiovascular death, and the ischemic events, such as myocardial infarction, repeat revascularization, TLR, TVR, and stent thrombosis, at a follow-up of longer than 5 years.

This study was conducted according to the principles expressed in the Declaration of Helsinki; all of the participants expressed oral informed consent. The study followed the Strengthening the Reporting of Observational Studies in Epidemiology (STROBE) reporting guidelines for cohort studies [9].

### Statistical Analysis

The baseline characteristics of the patients were represented as mean values with standard deviation (sd) and percentages. We performed multiple linear and logistic regression analyses to highlight potential clinical and procedural findings. Using a Kaplan–Meier survival analysis and Cox regression analysis, the occurrence of all-cause and cardiovascular death was also estimated as the risk of revascularization. Statistical significance was defined as 2-tailed *p* < 0.05. All statistical analyses were computed using the SPSS v.22 (IBM^®^ SPSS^®^ Statistics, 1 New Orchard Road Armonk, New York, NY, USA).

## 3. Results

**Figure 1:**
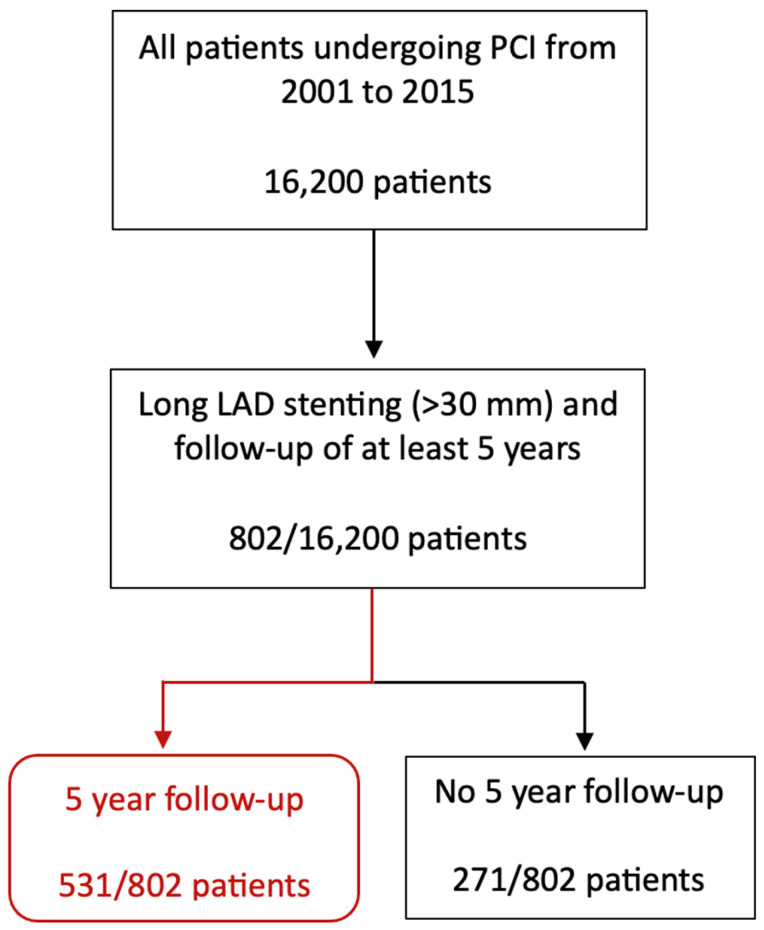
Patient inclusion flowchart for our study.

Five-hundred and thirty-one patients underwent PCI and the implantation of stents longer than 30 mm (Figure 1), with a FU longer than 60 months (5 years). The baseline characteristics are summarized in Table 1.

Procedural success was achieved in all of the procedures with implantations of stents longer than 30 mm; no intra-procedural deaths occurred. Patients who had died due to clinical events in the period before the 5 years of follow-up as a cut-off were included to collect mortality data. In terms of complications, three intra-procedural perforations occurred, and they were treated with long balloon inflation [2] or with a covered stent [1]; one acute vessel occlusion was treated with IIB/IIIA infusion and thrombus aspiration (Table 2).

The living patients were included in the study if they reached a minimum FU of 60 months (5 years). The clinical FU achieved a median of 92.18 ± 35.5 months. Angiographic FU was performed in 45.5% of the patients giving information about repeat revascularization (seven definite cases of stent thrombosis). During the FU, 97 patients died (18.3%), and among them, 56 (10.5%) died of cardiovascular disease. The data on the FUs and their outcome characteristics are shown in Table 3.

The rates of any incidences of MI, TLR, TVR, and repeat-revascularization were 9.3%, 14.5%, 16.9%, and 31.5%, respectively.

No statistically significant associations were found through the univariate and multivariate analyses combining the patients’ baseline and procedural characteristics with the outcomes.

Several statistical combinations, using Kaplan–Meier and Cox regression analyses, were performed in an attempt to identify a possible correlation between the baseline procedural features and technical-clinical outcomes. In Table 4, below, we summarize the statistically significant correlations.

The long-term follow-up surveys (mean 92.18 ± 35.5 months) showed a rate of all-cause death of 18.3% and of cardiovascular death of 10.5%. We found a statistically significant association between these results and those of three-vessel disease (HR 6.8; confidence of interval (CI) 95% 3.844–11.934; *p* < 0.001, and HR 4.7; CI 95% 2.265–9.835; *p* < 0.001, for all-cause death and cardiovascular death, respectively).

The myocardial infarction rate is 9.3% and it was not significantly related to any procedural or angiographic characteristics (Table 5 and Table 6). On the contrary, the three-lesion disease represented a risk factor for TLR and TVR (HR 3.4; CI 95% 1.984–5.781; *p* < 0.001; HR 3.9 CI 95% 2.323–6.442; *p* < 0.001, respectively).

Repeat revascularization (re-PCI) for any cause occurred in 31.4% of patients and shows an increased risk for the presence of three lesions (HR 4.3; CI 95% 2.873–6.376; *p* < 0.001) and the treatment of bifurcation treated with two stents (HR 1.6; 95% CI 1.051–2.414; *p* = 0.028).

The stent thrombosis rate at the 5-year FU was 4.4% (seven definite; five probable; eleven possible), including a 1.7% rate of very-late thrombosis, without any significant correlation to procedural characteristics.

We also investigated several ranges of stent lengths; in all of them, there were differences in mortality. We summarize the outcomes for the cut-off length of 40 mm in Table 5, comparing the population exposed and represented in the Kaplan–Meier survival curves in the central image (Graphic abstract).

The different segments of the LAD (proximal, mid, and distal) did not show any considerable correlations for the outcomes explored.

The presence of three lesions on the LAD as a marker of a long and diffuse disease showed a statistically significant increase in TLR risk (HR 3.4; CI 95% 1.984–5.781; *p* < 0.001), TVR (HR 3.9 CI 95% 2.323–6.442; *p* < 0.001) and re-PCI rate (HR 4.3; CI 95% 2.873–6.376; *p* < 0.001) (Table 6).

Furthermore, the re-PCI was significantly associated with the bifurcation treatment with two stents (HR 1.6; 95% CI 1.051–2.414; *p* = 0.028).

Finally, the rate of ST in our study was 4.3%, including acute ST (0.2%), sub-acute ST (0.6%), late ST (1.1%), and very-late ST (1.7%). Thanks to several angiographic FUs and a proper clinical FU, we could distinguish the definite ST (1.3%), probable ST (0.9%), and possible ST (2%). The ST was not statistically related to the procedural characteristics highlighted in the study (Table 5 and Table 6).

### Sensitivity Analysis on the Predictive Value of Missing Data

In consideration of the unexpected amount of patients lost at the FU (LFU), we performed a comparison analysis (Supplementary Materials) between the patients who accomplished long-term FUs (AFUs) and the LFU group; investigating the baseline and procedural characteristics revealed an equally high prevalence of male patients, with 84.3% vs. 85.6% (*p* = 0.775), respectively, in the AFU and LSU groups. The ages were significantly higher in the AFU group, with a mean age of 66 ± 11 vs. 63 ± 12.2 years old in the LFU group. Our comparison of the cases of hypertension, diabetes (with or without insulin therapy), hyperlipidemia, peripheral vascular disease, previous MI, CABG, left ventricle ejection fraction (LVEF), and the incidences of acute coronary syndromes did not show statistically significant differences (Supplementary Table S1). Among the baseline characteristics, re-PCI was performed in 38% of the AFU patients vs. 28.4% of the LFU group (*p* = 0.007).

Regarding the procedural data, the mean stent length (41.55 ± 12.8 mm in the AFU group vs. 39.97 ± 12 mm in the LFU group) and the mean stent diameter (3.09 ± 0.37 vs. 3.14 ± 0.38; *p* = 0.080) were similar in the two groups. No differences occurred between the two populations in relation to the prevalence of LAD ostial-proximal lesions, three-vessel disease, CTO, atherectomy, cutting balloon, IVUS/OCT use, perforation, IABP appliance, and intra-hospital MI.

The complex PCI was mostly represented in the AFU group (96.4% compared to 88.6% of the LFU group, *p* < 0.001), led by the three-lesion feature, the treatment of bifurcation with two stents, and the lesion type C (ACC/AHA classification). Finally, the percentage of post-dilatation performed on all of the stents implanted was 48% in the AFU vs. 63% in the LFU group (*p* < 0.001).

## 4. Discussion

The treatment of long and diffuse coronary lesions with percutaneous coronary intervention (PCI) has been problematic since the era of plain balloon angioplasty.

Diffuse lung lesions comprise more than 20% of contemporary clinical PCI practices and are a major determinant of unfavourable clinical outcomes.

Lesion length has been proven to be a factor related to higher rates of restenosis and TLR, and this risk is further increased by the multiplicity of implanted stents.

Even with DESs, diffuse diseases and long lesions are still associated with an increased risk of restenosis and the need for TLR and MACE.

However, there are limited data on the relative efficacy and safety of long-stented lesions and data on medium–long-term follow-ups are limited. Such information may have important clinical implications to help physicians select the optimal type of revascularization strategy, stenting modality, and follow-up strategies.

The long FU time of our multicentre, retrospective, observational, non-randomized study and the rigorous selection of our sample revealed considerable findings regarding the outcome of LAD treatment using long and very-long stents.

The overall mortality at a mean follow-up of longer than 90 months was 18.3%, while cardiovascular mortality was 10.5%; yet the demographic characteristics did not describe a population at severe risk. Less than one-third of the population was affected by diabetes, and the same rate applied for acute coronary syndrome.

The presence of complex angiographic characteristics contributed to the high rate of repeat revascularization (31.4%) and this is a peculiar finding of this study.

The limited use of physiology to evaluate lesions could explain the relatively high implantation rate of two stents in bifurcation lesions [10]. The high percentage of patients with DAPT could be more proof of the high ischemic risk of this population with diffuse atherosclerotic disease. Moreover, during the clinical FU, major bleeding events were rare and the DAPT was usually stopped after one year (mean 28.4 ± 33 months, median 11 months). It is probable that, in complex PCI, this standard DAPT interruption timing could be not optimal and could participate to a higher rate of re-PCI, instead of a longer and tailored regimen.

The stents used in our study were a mix of first-generation and basic-feature stents (BMSs, DESs, BVSs). More than 90% of the stents implanted were DESs, and 60% of them belonged to the second or the last DES generation. This could explain the good patency in the long term, even in this long stent setting, compared with the study on BMSs and first-generation DESs. A major long-term concern regarding DESs is the potential for stent thrombosis, which is increased after complex procedures involving the implantation of longer, multiple, and/or overlapping stents.

Our rate of repeat revascularization (31.4%) was slightly higher compared to the PCI results in the SYNTAX study at 5 years (25.9%); but, potentially, the PCI technical complexity of our study could lead to this difference (Table 7). At the same time, the MI incidence was similar to our PCI data (9.3% vs. 10.1% in the SYNTAX) [11,12]. Interestingly, in this comparison, we found a similar demographic risk profile concerning the population characteristics: around 20-25% were diabetics, 80% were male, and the average age was 66 in our study vs. 65 years old in the SYNTAX. However, even if we tried to correlate the high rate of re-PCI in our data to the clear angiographic complexity, the incidence of mortality does not follow this hypothesis, showing a low rate in the intermediate score that we could not compare due to the high technical patient selection of this registry.

Due to the considerable number of LFU patients, we compared this group with the study population in terms of baseline and procedural characteristics, investigating this potential bias through the predictors of poor outcomes.

This comparison highlighted a theoretically lower risk of poor events for the LFU group through a statistically significant difference in several baseline and procedural risk factors correlations. Indeed, the well-followed population showed a high prevalence of old age, previous PCI revascularizations, complex PCIs, three-lesion disease PCIs, bifurcations treated with two stents, lesions of type C, and the use of IABP. On the other hand, the group without FUs presented, as a speculative advantage, a high number of post-dilatation for all stents implanted, which should guarantee better stent apposition and fewer complications. The only discordant item in this disadvantageous trend for the main study population was a higher number of acute vessel occlusions. This mere speculation could be interpreted as justification and support for our data. The size of the LFU group, which departed moderately from the 25% used in similar very long-term studies, should characterize the least-possible outcome modifications. Of course, we could not determine the impact of the lost patients’ information, but we created a scenario of the expected results considering all the sample data available.

Although our study did not show the possible correlations with poor outcomes, our thoughts had to move with an educated and aware use of this extraordinary tool to assure the best treatment at the very-long FU.

Lesion length is still a convenient parameter for making clinical decisions and predicting outcomes, and it is reasonable that long stent implantation is more prone to lead to stent under-expansion, malposition, restenosis, and stent thrombosis [13]. However, lesion length per se—instead of being a true underlying reason for the higher ISR rate in the DES group—might merely be a surrogate for many other factors, such as the severity of disease, flow reserve, local inflammation, or lesion complexity. From this perspective, lesion length, even if associated with MACE-free survival, must always be contextualized according to the clinical complexity of the patient. In fact, in our results, it is not stent length that is associated with CV death but three-vessel disease, above all (see Table 4).

Our findings also reflect a non-contemporary era of PCI that is characterized by minimalist stent implantation techniques through physiology and imaging. The pharmacological background in terms of antiplatelet, hypolipidemic therapy and the treatment of hyperglycaemia is also constantly evolving with expected better outcomes [14,15].

## 5. Limitations

The present study had several limitations. Its major limitation is represented by the patients lost at the FU stage, which is 33.8%. We nevertheless tried to better understand the eventual impact of this population, comparing their characteristics with the followed-up patients.

Furthermore, this was an observational study and inevitably presented limitations in relation to its retrospective design. Although the commitment of the authors to limiting the multiple and natural confounding factors, we had to recognize that they still could have affected the study.

However, most of the limitations derived from the challenging patient selection; this study is the first real-world research, in our knowledge, to present results according to these procedural features of long stenting (30 mm inclusion criteria and investigation for the range of 40, 50, and 60 mm) at very long-term FUs.

Because of its observational nature, this study had to be considered as only a hypothesis-generating study, and the present findings need to be confirmed in larger and eventually prospective studies.

The population examined will also be subject to further follow-up to confirm the results even at a follow-up of longer than 5 years.

## 6. Conclusions

Patients adequately treated on their LAD with stents longer than 30 mm had acceptable clinical and procedural outcomes with an FU of longer than 5 years.

The higher risk seemed to be related to diffuse and multivessel diseases. It is our responsibility to improve, with passion, the treatment of percutaneous revascularization via the most innovative and physiological approach.

## Figures and Tables

**Table 1:** Baseline characteristics.

Male Sex (Total [%])	447/531 (84.3)
Mean Age, y	66 ± 11
Weight, kg	80 ± 58
Height, cm	166.5 ± 8.8
BMI, kg/m^2^	28.7 ± 18
Hypertension (%)	322/531 (60.6)
Diabetes (%)—on Insulin	140/531 (25.5)–39/531 (6.9)
Hyperlipidaemia (%)	330/531 (63.2)
Prior CABG (%)/Prior PCI (no LAD) (%)	61/531 (11.5)/202/531 (38)
Peripheral Vascular Disease (%)	44/531 (8.3)
Previous MI (%)	223/531 (42)
Stable Angina (%)	391/531 (73.7)
Unstable Angina (%)	9/531 (1.6)
STEMI/NSTEMI (%)	132/531 (24.8)

**Table 2:** Angiographic and procedural characteristics.

Mean Stent Length, *n* ± sd	41.5 ± 12.8 mm
Median stent length, mm	36
Minimal stent length, mm	31
Maximal stent length, mm	110
Mode stent length, mm	33
Mean stent diameter, mm ± sd	3.09 ± 0.37
LAD ostial-proximal, *n* (%)	189 (36)
Complex PCI features (ref. [8]), *n* (%)	512/531 (96.4)
3 Lesions, *n* (%)	256 (48.2)
3 Vessels, *n* (%)	167 (31.4)
CTO, *n* (%)	106 (20)
Bifurcation (2 stents), *n* (%)	128 (24.1)
Atherectomy, *n* (%)	40 (3.2)
Cutting balloon, *n* (%)	40 (7.5)
IVUS/OCT, *n* (%)	225 (42.3)
ACC/AHA C, *n* (%)	387 (72.8)
ACC/AHA B2, *n* (%)	134 (25.2)
ACC/AHA B1, *n* (%)	10 (1.9)
Post-dilatation on all stents, *n* (%)	255 (48)
Post-dilatation pressure > 19 atm	70 (20.1)
Perforation, *n* (%)	4 (0.7)
Acute vessel occlusion, *n* (%)	1 (0.2)
IABP, *n* (%)	41 (8)
Intra-hospital MI, *n* (%)	79 (15)

**Table 3:** Long-term FU (at least 5 years/60 months after PCI) and outcome characteristics.

Mean FU Length, *n* ± sd	92.18 ± 35.5 Months
Median of FU length	89 months
Patients after 5 years/60 months, *n* (%)	531 (100)
Patients after 7.5 years/90 months, *n* (%)	245 (46.1)
Patients after 10 years/120 months, *n* (%)	82 (15.4)
Stop DAPT from PCI average time	28.4 ± 33 months (median 11 months)
Patients on DAPT at clinical FU, *n* (%)	225 (42.4)
Death (all-cause), *n* (%)	97/531 (18.3)
CV * death, *n* (%)	56/531 (10.5)
Non-CV * death, *n* (%)	41/531 (7.7)
Any MI *, *n* (%)	49/531 (9.3)
TLR *, *n* (%)	77/531 (14.5)
TVR *, *n* (%)	90/531 (16.9)
Any re-PCI, *n* (%)	167/531 (31.4)
In-stent thrombosis, *n* (%)	23/531 (4.3)
acute stent thrombosis, *n* (%)	1 (0.2) (1 probable)
Sub-acute stent thrombosis, *n* (%)	3 (0.6) (3 possible)
Late stent thrombosis, *n* (%)	6 (1.1) (2 definite; 1 probable; 3 possible)
Very-late stent thrombosis, *n* (%)	9 (1.7) (5 definite; 1 probable; 7 possible)
Definite stent thrombosis, *n* (%)	7 (1.3)
Probable stent thrombosis, *n* (%)	5 (0.9)
Possible stent thrombosis, *n* (%)	11 (2)
Angiographic FU, *n* (%)	237/531 (45.5)
Angiographic FU average time	16.5 ± 22.7
Angiographic FU median time	8

* Abbreviations: CV = cardiovascular; MI = myocardial infarction; TLR = target lesion revascularization; TVR = target vessel revascularization.

**Table 4:** Statistically significant findings in the outcome investigations.

All-Cause Death	Statistical Correlation
3 Vessels	HR 6.8; CI 95% 3.844–11.934; *p* < 0.001
CV Death	
3 Vessels	HR 4.7; CI 95% 2.265–9.835; *p* < 0.001
Any Re-PCI	
3 Lesions	HR 4.3; CI * 95% 2.873–6.376; *p* < 0.001
Bifurcation	HR 1.6; CI * 95% 1.051–2.414; *p* = 0.028
TLR	
3 Lesions	RR 3.4; CI * 95% 1.984–5.781; *p* < 0.001
TVR	
3 Lesions	HR 3. 9; CI * 95% 2.323–6.442; *p* < 0.001

* CI: Confidence Interval.

**Table 5:** Stent Length.

Outcomes	Inferior or Equal to 40 mm 366/531	Superior to 40 mm 165/531	Statistic
All-cause death	63/366 (17.3%)	34/165 (20.6%)	*p* = 0.349
Cardiovascular death	38/366 (10.4%)	18/165 (10.9%)	*p* = 0.855
MI	35/366 (9.5%)	14/165 (8.5%)	*p* = 0.691
Re-PCI	121/366 (33%)	46/165 (27.8%)	*p* = 0.234
TLR	54/366 (14.7%)	23/165 (13.9%)	*p* = 0.805
TVR	69/366 (18.8%)	21/165 (12.7%)	*p* = 0.087
Stent thrombosis	13/366 (3.6%)	10/165 (6%)	*p* = 0.189

**Table 6:** Three lesions treated on LADs.

Outcomes	Less Than 3 275/531	3 or More 256/531	Statistic
All-cause death	63/275 (22.9%)	34/256 (13.3%)	*p* = 0.146
Cardiovascular death	29/275 (10.5%)	27/256 (10.5%)	*p* = 0.250
MI	21/275 (7.6%)	18/256 (7%)	*p* = 0.092
Re-PCI	47/275 (17%)	120/256 (46.8%)	*p* < 0.001
TLR	21/275 (7.6%)	56/256 (14.3%)	*p* < 0.001
TVR	23/275 (8.3%)	67/256 (17.1%)	*p* < 0.001
Stent thrombosis	13/275 (4.7%)	10/256 (3.9%)	*p* = 0.642

**Table 7:** Comparison of our outcomes with those of the SYNTAX study.

	Overall Mortality	* CV Mortality	MI	* Re-Revasc.	ST Def–Prob	TLR	TVR
Our whole population	97/531 (18.3)	56/531 (10.5)	49/531 (9.3)	167/531 (31.4)	12/531 (1.2)	77/531 (14.5)	90/531 (16.9)
Stent length 30–40	63/366 (17.3)	38/366 (10.4)	35/366 (9.5)	121/366 (33)	5/366 (1.4)	54/366 (14.7)	69/366 (18.8)
Stent length > 40 mm	34/165 (20.6)	18/165 (10.9)	14/165 (8.5)	46/165 (27.8)	7/165 (4.2)	23/165 (13.9)	21/165 (12.7)
SYNTAX study, intermediate score	42/310 (13.8)	26/310 (8.8)	33/310 (11.2)	70/310 (24.1)	47/708 (6.6)		
SYNTAX study, high score	55/290 (19.2)	38/290 (13.6)	28/290 (10.1)	83/290 (30.9)			

Numbers illustrated as n/total (%). * CV = cardiovascular; Re-Revasc = repeat revascularization; MI = myocardial infarction; ST Def–Prob = stent thrombosis, definite/probable.

## Data Availability

Not available due to privacy policy of the institutions involved.

## Supplementary Materials

The following supporting information can be downloaded at https://www.mdpi.com/article/10.3390/jcm13010210/s1: Complete comparison of baseline and procedural characteristics of patients with and without accomplished FU. Complete procedural data.

## Abbreviations

Follow-up (FU); percutaneous coronary intervention (PCI); left anterior descending artery (LAD); follow-up (FU); myocardial infarction (MI); target vessel revascularization (TVR); target lesion revascularization (TLR); repeat percutaneous coronary intervention (re-PCI); stent thrombosis (ST); hazard ration (HR); confidence of interval (CI); coronary bypass surgery (CABG); left internal mammary (LIMA); left ventricle ejection fraction (LVEF); coronary total occlusion; intra-stent restenosis (ISR); standard deviation (sd); lost at FU (LFU); accomplished long-term FU (AFU); bare metal stent (BMS); drug-eluting stent (DES); bioresorbable vascular stent (BVS).
