# Supplementary material for: Long Stent Implantation on the Left Anterior Descending Coronary Artery at a Follow-Up of More Than Five Years
Source: J Clin Med. 2023 Dec 29;13(1):210. doi: 10.3390/jcm13010210 (PMC10779530; doi:10.3390/jcm13010210)
Supplement: Supplementary file 1 [file jcm-13-00210-s001.zip › jcm-2687480-supplementary.pdf]

Supplementary Material

Table S1. Complete comparison of baseline and procedural characteristics of patients with and without accomplished FU.

**Characteristic**	**Group with FU (n 531/802; 66%)**	**Without FU (n 271/802; 34%)**	Statistic
Male sex/total (%)	447/531 (84.3)	148/271 (85.6)	p=0.775
Mean Age, y	66 ± 11	63 ± 12.2	p<0.001
Hypertension (%)	322/531 (60.6)	155/271 (57.2)	p=0.384
Diabetes (%)	140/531 (25.5)	58/271 (21.4)	p=0.123
On Insulin	39/531 (6.9)	12/271 (4.4)	
Hyperlipidaemia (%)	330/531 (63.2)	158/271 (58.3)	p=0.292
Prior CABG (%)	61/531 (11.5)	15/271 (5.5)	p=0.060
Prior PCI (%)	202/531 (38)	77/271 (28.4)	p=0.007
Peripheral Vascular Disease (%)	44/531 (8.3)	17/271 (6.3)	p=0.310
Previous MI (%)	223/531 (42)	117/271 (43.2)	p=0.712
LVEF %	53.1	54.5	p=0.230
Stable Angina (%)	391/531 (73.7)	216/271 (79.7)	p=0.580
Unstable Angina (%)	9/531 (1.6)	2/271 (0.8)	p=0.271
STEMI/NSTEMI (%)	132/531 (24.8)	53/271 (19.5)	p=0.920
**Complete Procedural Data**			
Mean Stent Length, n ± sd	41.5 ± 12.8 mm	39.97 ± 12 mm	p=0.182

Median Stent Length	36	33	
Minimal Stent Length	31	31	
Maximal Stent Length	110	99	
Mode Stent Length	33	33	
Mean Stent Diameter	3.09 ± 0.37	3.14 ± 0.38	p=0.080
LAD ostial-proximal, n (%)	189 (36)	110 (40.5)	p=0.532
Complex PCI Features [ref. (94)], n (%)	512 (96.4)	240 (88.6)	p<0.001
3 Lesions, n (%)	256 (48.2)	202 (74.5)	p<0.001
3 Vessels, n (%)	167 (31.4)	52 (19.2)	p=0.607
CTO, n (%)	106 (20)	38 (14)	p=0.380
Bifurcation (2 stents), n (%)	128 (24.1)	96 (35.5)	p<0.001
Atherectomy, n (%)	40 (3.2)	14 (5.2)	p=0.173
Cutting Balloon, n (%)	40 (7.5)	25 (9.2)	p=0.407
IVUS/OCT, n (%)	225 (42.3)	133 (49.1)	p=0.710
ACC/AHA C, n (%)	387 (72.8)	146 (53.8)	p<0.001
Post-dilatation on all stents n (%)	255 (48)	170 (63)	p<0.001
Perforation, n (%)	4 (0.7)	0 (0)	p=0.152
Acute vessel occlusion, n (%)	1 (0.2)	4 (1.5)	p=0.028
IABP, n (%)	41 (8)	8 (2.9)	p=0.080
Intra-hospital MI, n (%)	79 (15)	33 (12.2)	p=0.297
